# Fully covered self‐expandable metallic stents versus plastic stents for preoperative biliary drainage in patients with pancreatic head cancer and the risk factors for post‐endoscopic retrograde cholangiopancreatography pancreatitis

**DOI:** 10.1002/deo2.263

**Published:** 2023-06-26

**Authors:** Fumisato Kozakai, Takahisa Ogawa, Sinsuke Koshita, Yoshihide Kanno, Hiroaki Kusunose, Toshitaka Sakai, Keisuke Yonamine, Kazuaki Miyamoto, Hideyuki Anan, Haruka Okano, Kento Hosokawa, Kei Ito

**Affiliations:** ^1^ Department of Gastroenterology Sendai City Medical Center Miyagi Japan

**Keywords:** pancreatic cancer, preoperative biliary drainage, obstructive jaundice, self‐expandable metallic stent, surgery

## Abstract

**Objectives:**

Optimal stents for preoperative biliary drainage (PBD) for patients with possible resectable pancreatic cancer remain controversial, and risk factors for post‐endoscopic retrograde cholangiopancreatography pancreatitis (PEP), followed by PBD, are unknown. In this study, the efficacy and safety of fully covered self‐expandable metallic stents (FCSEMSs) and plastic stents (PSs) were compared, and the risk factors for PEP, followed by PBD, were investigated for patients with pancreatic cancer.

**Methods:**

Consecutive patients with pancreatic cancer who underwent PBD between April 2005 and March 2022 were included. We retrospectively evaluated recurrent biliary obstruction, adverse events (AEs), and postoperative complications for FCSEMS and PS groups and investigated the risk factors for PEP.

**Results:**

A total of 105 patients were included. There were 20 patients in the FCSEMS group and 85 patients in the PS group. For the FCSEMS group, the rate of recurrent biliary obstruction (0% vs. 25%, *p* = 0.03) was significantly lower. There was no difference in AE between the two groups. No significant differences were observed in the overall postoperative complications, but the volume of intraoperative bleeding was larger for the PS group than it was for the FCSEMS group (*p* < 0.001). From multivariate analysis, being female and lack of main pancreatic duct dilation were independent risk factors for pancreatitis (odds ratio, 5.68; *p* = 0.028; odds ratio, 4.91; *p* = 0.048).

**Conclusions:**

FCSEMSs are thought to be preferable to PSs for PBD due to their longer time to recurrent biliary obstruction. Being female and the lack of main pancreatic duct dilation were risk factors for PEP.

## INTRODUCTION

So far, surgery has been the only curative treatment for pancreatic cancer (PC). However, the prognoses for patients with PC have been very poor even in resected cases because postoperative recurrence due to positive resected margins is very common.^1^, ^2^ Recently, several studies have reported that neoadjuvant chemotherapy (NAC) for borderline resectable PCs (BRPCs) can improve the R0 resection rate and prognosis.^3^, ^4^, ^5^, ^6^ In addition, from more recent studies, NAC can be effective not only for BRPC cases but also for resectable PC (RPC) cases.^7^, ^8^ Therefore, NAC is becoming standard for possible resectable PCs.

Pancreatic head cancer is often associated with obstructive jaundice. In a few studies, it has been suggested that preoperative biliary drainage (PBD) is not indispensable for all preoperative cases.^9^, ^10^, ^11^ However, it is necessary to perform PBD to administer NAC.

For palliative biliary drainage of unresectable cases, self‐expandable metallic stents (SEMSs) have been recommended since their stent patencies have been reported to be longer than those of plastic stents (PSs) in several studies.^12^, ^13^ On the other hand, for PBD, the choice of the optimal stent remains controversial. SEMSs are thought to be preferable for cases where the wait time for surgery is relatively long, for example, NAC cases (the wait time for surgery is 3–6 months).^14^, ^15^, ^16^, ^17^, ^18^, ^19^, ^20^, ^21^, ^22^, ^23^, ^24^ However, from meta‐analyses comparing SEMSs with PSs for PBDs, the rate of post‐endoscopic retrograde cholangiopancreatography pancreatitis (PEP) has been reported to be higher for patients receiving SEMSs than that for those receiving PSs.^25^, ^26^ PEP can interfere with the beginning of NAC and cause adhesion around the pancreas causing difficulties during surgery.

There have been no studies regarding risk factors for PEP, followed by PBD, for patients with PCs. Therefore, we evaluated the efficacy and safety of SEMS and PS and investigated the risk factors for PEP, followed by PBD, for patients with PC.

## PATIENTS AND METHODS

### Patients

This was a single‐center retrospective observational study. Consecutive patients with pancreatic head cancer who underwent PBD at our medical center between April 2005 and March 2022 were included in this study. The following patients were excluded: 1) patients who underwent percutaneous transhepatic biliary drainage and endoscopic ultrasound‐guided biliary drainage (EUS‐BD), 2) those who underwent endoscopic nasobiliary drainage, 3) those who did not undergo initial drainage (patients who had undergone drainage at other hospitals), and 4) those who refused to participate in the study. Unresected cases (becoming unresectable or refusing surgery after PBD) were not excluded from the study.

This study was approved by the institutional review board of Sendai City Medical Center (approval number, 2019‐0035). Written informed consent for the procedures was obtained from all patients.

### Confirmation of diagnosis

All patients underwent multi‐detector‐low computed tomography, magnetic resonance cholangiopancreatography, or EUS to evaluate the resectability before PBD. The diagnosis between RPC and BRPC was made following the National Comprehensive Cancer Network guidelines.^27^ Histological confirmation of PC was performed by using either EUS‐guided fine needle aspiration, a biopsy from the site of invasion of PC (duodenum or/and bile duct), or pancreatic juice cytology. For cases in which histopathological confirmation was impossible before surgery, it was performed using histopathological examinations of resected specimens.

### Intervention

PBD was performed using JF260V, TJF260V, or TJF‐Q290V duodenoscopes (Olympus CO., Tokyo, Japan) under moderate sedation with intravenous pentazocine and midazolam. Before PBD, endoscopic sphincterotomy was performed for all patients, and biopsies from the biliary stricture and/or pancreatic juice cytology were performed at the discretion of the endoscopist. A fully covered SEMS (FCSEMS) or a PS was placed across the duodenal papilla for PBD. FCSEMSs were mainly used later in the observational period. The selection of FCSEMSs was determined by the operator on the basis of the necessity for NAC or the waiting time for surgery. The hepatic end of the FCSEMS was located at least 10 mm below the bifurcation of the hepatic duct. FCSEMS used were as follows: BONA STENT Biliary with Lasso (Medico's Hirata Inc., Tokyo, Japan) for 12 patients, HANAROSTENT Biliary (Boston Scientific Japan K.K., Tokyo, Japan) for 5, WallFlex Biliary RX Fully Covered Stents (Boston Scientific Japan K.K., Tokyo, Japan) for two, and Evolution Biliary Controlled‐Release Stent‐Fully Covered (Cook Medical Japan, Tokyo, Japan) for one. The diameters of the FCSEMSs used were 8 or 10 mm. The diameters of the PSs used ranged from 7‐ to 11.5‐Fr.

### Neoadjuvant chemotherapy

The decision to perform NAC for each patient was made by the surgeons.

The NAC protocol for RPC cases was as follows: three cycles of gemcitabine/S‐1 combination therapy (gemcitabine 1000 mg/m^2^, on days 1 and 8 every 3 weeks, S‐1 80, 100, and 120 mg/body/day, on days 1–14 every 3 weeks).

NAC protocol for BRPC was as follows: 2 cycles of gemcitabine/nab‐paclitaxel combination therapy (gemcitabine 1000 mg/m^2^, nab‐paclitaxel 125 mg/m^2^, on days 1, 8, and 15 every 4 weeks).

The efficacy of NAC was evaluated by using multi‐detector‐low computed tomography findings and variations in the tumor marker levels. The indication for surgery was determined after discussion among the surgeons on the basis of the NAC efficacy.

### Outcome measurements and definitions

**FIGURE 1:**
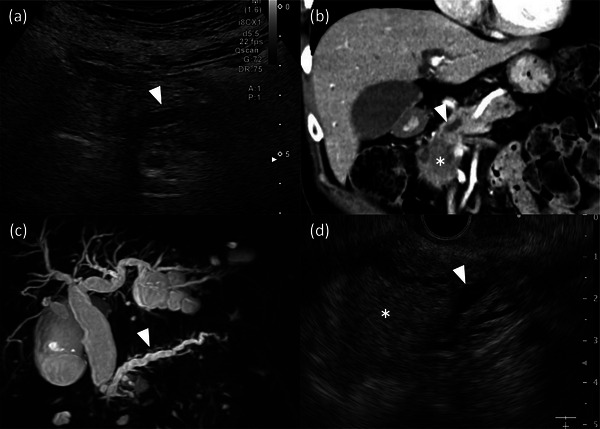
Image findings of the main pancreatic duct dilation (MPD Dil.) (a) abdominal ultrasonography findings MPD Dil. (arrowhead, MPD Dil.). (b) CT scan showing MPD Dil. because of stenosis with pancreatic head cancer: Arrowhead, MPD Dil.; asterisk, cancer. (c) Magnetic resonance cholangiopancreatography findings showing MPD Dil.: Arrowhead, MPD Dil. (d) EUS findings showing MPD Dil. caudal to cancer: arrowhead, MPD Dil.; asterisk, cancer. Cases with any of these findings were considered to be MPD Dil.

The outcome measurements were clinical success, recurrent biliary obstruction (RBO), time to RBO (TRBO), adverse events (AEs) other than RBO, postoperative complications, and risk factors for PEP. Clinical success was defined as a decrease in the bilirubin level to normal or <50% of the levels within 14 days after biliary stenting. RBO was defined as stent occlusion and/or migration, and TRBO was defined as the period from stent placement to RBO. Patients who could not undergo curative resection or who refused surgery after PBD were treated as censored cases on the operation day. AEs, other than RBO, were assessed on the basis of consensus criteria^28^ and categorized as early (⩽30 days after stenting) and late (≥31 days after stenting). Postoperative complications were evaluated according to the Clavien‐Dindo classification^29^ within 90 days of surgery. Main pancreatic duct (MPD) dilation was defined as a diameter of MPD thicker than 3 mm measured by using either abdominal ultrasonography, EUS, multi‐detector‐low computed tomography, or magnetic resonance cholangiopancreatography (Figure 1).

### Statistical analyses

Continuous variables were assessed using a Mann‐Whitney U test or Kruskal‐Wallis test, and categorical variables were assessed using a chi‐square test or Fisher exact test. A *p*‐value of <0.05 was considered to be statistically significant. TRBO was analyzed using the Kaplan‐Meier method and a log‐rank test. The risk factors for PEP were evaluated using univariate and multivariate analyses. The multivariate analyses were performed using logistic regression analysis referencing the risk factors reported in previous studies,^30^, ^31^ and the results are presented with a hazard ratio and 95% confidence intervals (CIs). The SPSS software program (version 24; IBM Japan, Ltd., Tokyo, Japan) was used for the analyses.

## RESULTS

### Patient characteristics

**FIGURE 2:**
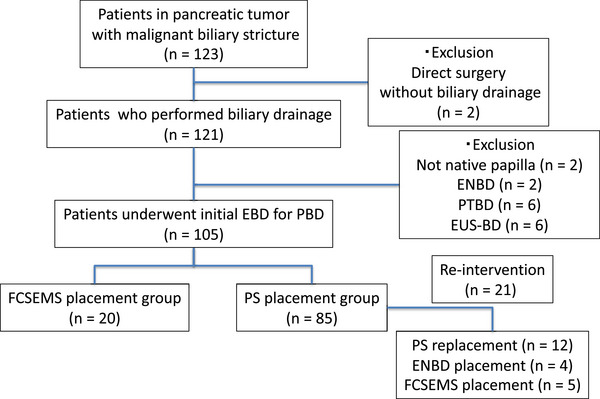
Flowchart of clinical course in this study. PTBD, percutaneous transhepatic biliary drainage; ENBD, endoscopic nasobiliary drainage; EUS‐BD, endoscopic ultrasound‐guided biliary drainage; EBD, endoscopic biliary drainage; PBD, preoperative biliary drainage; FCSEMS, fully covered self‐expandable metallic stent; PS, plastic stent.

One hundred and twenty‐three consecutive patients diagnosed with RBC/BRPC underwent PBD at our center between April 2005 and March 2022. Among them, 18 patients who did not undergo FCSEMS or PS placement as the initial drainage were excluded. Finally, 105 patients were included in this study (Figure 2).

The patient characteristics are shown in Table 1. FCSEMSs were placed for 20 patients (FCSEMS group), and PSs were placed for 85 patients (PS group). In the PS group, 10‐Fr or greater PSs were used for 53 patients (62%; ≥10‐Fr PS group). For the remaining 32 patients (48%), <10‐Fr PSs were used (8.5‐Fr for 23 patients and 7‐Fr for nine patients; <10‐Fr PS group). For one patient in the ≥10‐Fr PS group and for three patients in the <10‐Fr PS group, two PSs were placed(Table 2). Twenty‐nine patients (28%) received NAC. The number of NAC patients was significantly higher for the FCSEMS group, and therefore the waiting time for surgery was significantly longer for the FCSEMS group. There was no difference in the proportion of RPC between the two groups (80% [16/20] vs. 84% [71/85]; *p* = 0.96). The percentages of the presence of MPD dilation were similar for the two groups (85% [17/20] vs. 71% [60/85]; *p* = 0.30).

**TABLE 1:** Baseline characteristics of the patients.

	FCSEMS group (*n* = 20)	PS group (*n* = 85)	*p‐*Value
Patient characteristics			
Age, years (mean ± SD)	67 ± 11	68 ± 9.6	0.71
Male:female	14:6	55:30	0.65
Total bilirubin, mg/dl (mean ± SD)	8.2 ± 4.7	9.0 ± 6.8	0.54
NAC, % (*n*)	55 (11)	21 (18)	0.002
Waiting time to the surgery, days (mean ± SD)	71 ± 46	48 ± 42	0.033
Tumor size (≤ 20 mm), % (*n*)	10 (2)	42 (36)	0.014
Main pancreatic duct dilation, % (*n*)	85 (17)	71 (60)	0.30
Resectable:borderline resectable	16:4	71:14	0.96
Administration of rectal NSAIDs, % (*n*)	75 (15)	25 (21)	<0.001
Tumor characteristics, % (*n*)			0.10
Adenocarcinoma	80 (16)	92 (78)	
IPMC	10 (2)	7 (6)	
Anaplastic carcionoma	0 (0)	1 (1)	
Neuroendocrine carcinoma	5 (1)	0 (0)	
Adenosquamous carcinoma	5 (1)	0 (0)	

Abbreviations: FCSEMS, fully covered self‐expandable metallic stent; IPMC, intraductal papillary mucinous neoplasm with an associated invasive carcinoma; NAC, neoadjuvant chemotherapy; NSAIDs nonsteroidal anti‐inflammatory drugs; PS, plastic stent.

**TABLE 2:** Features of biliary stents.

	FCSEMS group (*n* = 20)	PS group (*n* = 85)
Number of stents, % (*n*)		
One	100 (20)	95 (81)
Two	0 (0)	5 (4)
Stent diameter, % (*n*)		
10 mm	90 (18)	
8 mm	10 (2)	
11.5‐Fr		2 (2)
10‐Fr		59 (50)
8.5‐Fr		24 (20)
7‐Fr		11 (9)
10‐Fr and 7‐Fr		1 (1)
8.5‐Fr and 7‐Fr		3 (3)

Abbreviations: FCSEMS, fully covered self‐expandable metallic stent; PS, plastic stent.

### Clinical success and RBO

**FIGURE 3:**
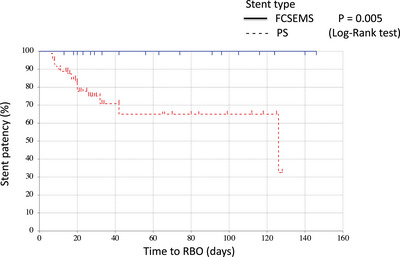
Kaplan‐Meier analysis of cumulative stent patency. The median time to recurrent biliary obstruction (RBO) for the fully covered self‐expandable metallic stent group was not reached, whereas the plastic stent group had a mean patency of 126 days (p = 0.005, log‐rank test).

**FIGURE 4:**
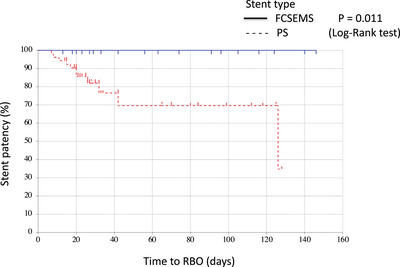
Kaplan‐Meier analysis of cumulative stent patency. Time to recurrent biliary obstruction (RBO) for the plastic stent (PS) group (≥10Fr) had a median patency of 126 days (p = 0.011, log‐rank test). FCSEMS, fully covered self‐expandable metallic stent.

The outcomes are shown in Table 3. Clinical success rates were 100% for the FCSEMS group and 82% for the PS group (*p* = 0.09). The 15 clinically unsuccessful patients underwent re‐intervention (exchange of the stent (PS to FCSEMS for three patients and to a larger PS for one patient) and endoscopic nasobiliary drainage for two patients). The rate of RBO was significantly lower for the FCSEMS group than it was for the PS group (0% [0/20] vs. 25% [21/85]; *p* = 0.030). TRBO was significantly longer for the FCSMES group. The median TRBO was 126 days for the PS group, although it did not reach the median value during the observation period for the FCSMES group. The patency rate at three months from stent placement was significantly higher for the FCSEMS group (100% vs. 60%; *p* = 0.005; Figure 3). TRBO was significantly shorter for the ≥10‐Fr PS group than that for the FCSEMS group (*p* = 0.011; Figure 4).

**TABLE 3:** Outcomes of endoscopic biliary drainage.

	FCSEMS group (*n* = 20)	PS group (*n* = 85)	*p*‐Value
Clinical success, % (*n*)	100 (20)	82 (70)	0.09
RBO, % (*n*)	0 (0)	25 (21)	0.030
Time to RBO, days (median)	Nor reached	126	0.005
Adverse events other than RBO, % (*n*)			
Early (≤ 30 days)	30 (6)	27 (23)	0.79
Post‐ERCP pancreatitis	20 (4)	8.2 (7)	0.25
(mild/moderate/severe)	(1/2/1)	(4/2/1)	
Acute cholangitis	10 (2)	16 (14)	0.70
Acute cholecystitis	0 (0)	3.5 (3)	0.39
Bleeding	0 (0)	1.2 (1)	0.63
Late (≥31 days)	10 (2)	4.7 (4)	0.36
Acute pancreatitis	5 (1)	0 (0)	0.038
Acute cholangitis	5 (1)	2.4 (2)	0.52
Acute cholecystitis	0 (0)	1.2 (1)	0.63
Liver abscess	0 (0)	1.2 (1)	0.63
Total	35 (7)	36 (31)	0.90

Abbreviations: FCSEMS, fully covered self‐expandable metallic stent; ERCP, endoscopic retrograde cholangiopancreatography; RBO, recurrent biliary obstruction; PS, plastic stent.

### Adverse events

Early AEs were not significantly different between the two groups (30% [6/20] vs. 27% [23/85]; *p* = 0.79). The incidence of PEP was 20% for the FCSEMS group. However, there was no significant difference in comparison to the PS group (20% [4/20] vs. 8.2% [7/85]; *p* = 0.25). All patients improved after only conservative treatment. Late AEs were similar for both groups (10% [2/20] vs. 4.7% [4/85]; *p* = 0.36).

### Postoperative complications

Fifteen patients did not undergo resection because their cancers were judged to be unresectable intraoperatively or they refused surgery after PBD. The remaining 90 patients (13 in the FCSEMS group and 77 in the PS group) underwent pancreatoduodenectomy. There were no differences in postoperative complications (23% (3/13) vs. 42% (32/77); *p* = 0.44) between the two groups. The median volume of intraoperative bleeding was significantly less for the FCSEMS group than it was for the PS group (753 ml vs. 1122 ml; *p* < 0.001; Table 4).

**TABLE 4:** Outcomes after surgical procedure.

	FCSEMS group (*n* = 13)	PS group (*n* = 77)	*p*‐Value
Surgical procedure, % (*n*)			0.58
SSPPD	92 (12)	86 (66)	
PD	0 (0)	13 (10)	
TP	8 (1)	0 (0)	
PD + TG	0 (0)	1 (1)	
Operation time, min (mean ± SD)	426 ± 48	440 ± 63	0.38
Postoperative hospitalization, days (mean ± SD)	31 ± 28	32 ± 13	0.85
Volume of intraoperative bleeding (ml), median (range)	753 (470–1050)	1122 (178–3360)	<0.001
Adverse events, % (*n*)	23 (3)	42 (32)	0.44
Clavien‐Dindo gradeII			
Pancreatic fistula	0 (0)	3 (2)	0.34
Delayed gastric emptying	8 (1)	8 (6)	0.34
Cholangitis	0 (0)	9 (7)	0.57
Thrombosis	0 (0)	5 (4)	0.40
Others (*n*)	0	9*	N/A
Clavien‐Dindo ≥gradeIIIa			
Pancreatic fistula	8 (1)	18 (14)	0.59
Others (*n*)	1^⁑^	7^#^	N/A

Abbreviations: PD, pancreaticoduodenectomy; SSPPD, subtotal stomach‐preserving pancreatoduodenectomy; TP, total pancreatectomy.

^*^ Surgical site infection (SSI), Pneumonia, arrhythmia, ileus, Liver abscess. It contains duplicate cases.

^⁑^ Pleural effusion.

^#^ Intra‐abdominal abscess, SSI, Anastomotic hemorrhage, Pneumonia (grade IVa), Ventricular arrhythmia (grade V), It contains duplicate cases.

### Risk factors for PEP

The results from univariate and multivariate analyses for risk factors for PEP are shown in Table 5. From univariate analyses, being female and biliary biopsy were predictors for an increase in the occurrence of PEP (*p* = 0.040, *p* = 0.033). From multivariate analysis, being female and the lack of MPD dilation were independent risk factors for PEP (odds ratio, 5.68; 95% CI 1.20–26.9; *p* = 0.028 and odds ratio, 4.91; 95% CI 1.02–23.7; *p* = 0.048). The FCSEMS was not an independent risk factor for PEP (*p* = 0.173).

**TABLE 5:** Risk factors for post‐endoscopic retrograde cholangiopancreatography pancreatitis after preoperative biliary drainage (PBD).

		Univariate analysis	Multivariate analysis	
		*p‐*‐Value	*p*‐Value	OR (95% CI)
Gender	Female	0.040	0.028	5.68 (1.20–26.9)
Age	⩽64 years	0.836		
Resectable	Yes	0.998		
MPD Dil.	Lack	0.147	0.048	4.91 (1.02–23.7)
Tumor size	<20 mm	0.990		
Tumor size	<20 mm	0.990		
Duodenal stenosis	Yes	0.195		
Total bilirubin	>8 mg/dl	0.725		
Administration of rectal NSAIDs	No	0.145	0.730	
Stent type	FCSMS	0.134	0.173	
Pancreatography	Yes	0.513		
EST	No	0.999		
Precut	Yes	0.129		
Pancreatic stenting	Yes	0.051	0.088	
IDUS	Yes	0.938		
Biliary biopsy	Yes	0.033	0.147	
Procedure time	≥30 min	0.646		

Abbreviations: CI, confidence interval; FCSEMS, fully covered self‐expandable metallic stent; IDUS, intraductal ultrasonography; MPD Dil., main pancreatic duct dilation; NSAIDs, nonsteroidal anti‐inflammatory drugs; OR, odds ratio; PEP, post‐endoscopic retrograde cholangiopancreatography pancreatitis.

## DISCUSSION

Although FCSEMSs for PBD in patients with PCs have advantages in terms of TRBO and RBO rate compared to PSs,^23^, ^32^, ^33^, ^34^ PEP is a concern when using FCSEMSs^25^, ^26^ due to potential blocking of the MPD orifice. Indeed, in two published meta‐analyses, it has been reported that the rate of PEP is higher for the FCSEMS group than it is for the PS group.^25^, ^26^ In this study, although PEP occurred more frequently in the FCSEMS group (20% vs. 8.2%) than it did in the PS group, there was no significant difference between the two groups.

There are several studies in which the risk factors for PEP were investigated.^30^, ^31^ However, there have been no reports in which only PBD cases have been investigated. This is the first study where the risk factors for PEP only in the case of PBD have been investigated. The results of our study showed that the use of FCSEMS was not a risk factor for PEP, but that being female and the lack of MPD dilation were independent risk factors for PEP.

Being female and the lack of MPD dilation have previously been reported to be risk factors for PEP in published studies.^30^, ^31^ Ding et al.^30^ have reported a meta‐analysis from which the risk factors for PEP are female gender, previous pancreatitis, previous PEP, sphincter of Oddi dysfunction, intraductal papillary mucinous neoplasm, difficult cannulation, endoscopic sphincterotomy, precut sphincterotomy, and main pancreatic duct injection. More recently, Xia et al have investigated the risk factors for PEP after FCSEMS placement in 602 patients with biliary strictures, including both benign and malignant cases, and they have reported that bile duct canulation without pancreatic stent placement and the lack of MPD dilation are risk factors for PEP.^31^ For many patients with pancreatic head cancer, MPD dilation is when the stent becomes occluded due to tumor invasion. Since the caudal pancreatic parenchyma atrophies in such patients, pancreatic exocrine function deteriorates, and the effects due to blocking of the MPD orifice with FCSEMS become small. On the other hand, the pancreatic exocrine function of the patients without MPD dilation is maintained because caudal pancreatic parenchyma does not atrophy when there is no MPD obstruction. For such patients, blockage of the MPD orifice because the FCSEMS tends to trigger PEP.^35^, ^36^

There were no significant differences regarding postoperative complications between the two groups in our study. This result is similar to recent meta‐analyses.^26^, ^37^ The amount of intraoperative bleeding was significantly higher for the PS group than it was for the FCSEMS group in our study. However, in an RCT comparing FCSEMSs with PSs for PBD, Mandai et al. reported a significant amount of intraoperative bleeding for the FCSEMS group.^38^ All patients included in their study did not undergo NAC. On the other hand, in our study, NAC was performed for about half of the patients in the FCSEMS group. Concerning a lower amount of intraoperative bleeding, the efficacy of chemotherapy may influence the ease of surgery.

In terms of RBO and TRBO, the results were significantly better for the FCSEMS group and similar to recent previous reports.^21^, ^22^, ^23^ In addition, it was confirmed that the TRBOs for PSs were not sufficient to fully carry out NAC. The diameters of the PSs used in this study ranged from 7‐ to 11.5‐Fr. Theoretically, a thick PS has a longer TRBO than a thin PS. Therefore, we performed a subgroup analysis comparing the ≥10‐Fr PS group with the FCSEMS group. However, TRBO was significantly shorter for the ≥10‐Fr PS group than it was for the FCSEMS group. Acute cholangitis due to stent occlusion while waiting for surgery has been reported to be associated with postoperative complications.^24^, ^39^ Therefore, FCSEMSs are often preferable for cases where the waiting time for surgery is relatively long, like NAC cases. In cases of not only being male but also MPD dilation, placement of an FCSEMS could be recommended. However, there is concern about the high risk for PEP in the FCSEMS group. Thus, if the patients have risk factors for PEP, scheduled PS replacement would be desired for PBD.

Our study has several limitations. First, this was a single‐center, retrospective study. Second, the sample size of the FCSEMS group was small. Therefore, our results showing that FCSEMSs were not a risk factor for PEP could be a type II error. In addition, there were statistical concerns about multivariate analysis of the risk factors for PEP because the number of PEP occurrences was small. However, being female and the lack of MPD dilation was shown to be independent risk factors for PEP. From our results, FCSEMSs appear to be better for patients who do not have such risk factors. Third, the patients who did not receive NAC were also included.

In conclusion, FCSEMSs are preferable to PSs in the case of PBD for patients with RPC/BRPC due to their longer TRBOs. Being female and the lack of MPD dilation were risk factors for PEP, but the use of FCSEMSs was not.

## CONFLICT OF INTEREST STATEMENT

The authors declare no conflict of interest.
